# Synostosis Between Pubic Bones due to Neurogenic, Heterotopic Ossification

**DOI:** 10.1100/tsw.2006.387

**Published:** 2006-10-02

**Authors:** Subramanian Vaidyanathan, Peter L. Hughes, Bakul M. Soni

**Affiliations:** ^1^ Regional Spinal Injuries Centre, District General Hospital, Southport, PR8 6PN, UK; ^2^ Department of Radiology, District General Hospital, Southport, PR8 6PN, UK

**Keywords:** synostosis, heterotopic ossification, spina bifida, urine, symphysis pubis, suprapubic cystostomy, diastasis of pubis

## Abstract

Neurogenic, heterotopic ossification is characterised by the formation of new, extraosseous (ectopic) bone in soft tissue in patients with neurological disorders. A 33-year-old female, who was born with spina bifida, paraplegia, and diastasis of symphysis pubis, had indwelling urethral catheter drainage and was using oxybutynin bladder instillations. She was prescribed diuretic for swelling of feet, which aggravated bypassing of catheter. Hence, suprapubic cystostomy was performed. Despite anticholinergic therapy, there was chronic urine leak around the suprapubic catheter and per urethra. Therefore, the urethra was mobilised and closed. After closure of the urethra, there was no urine leak from the urethra, but urine leak persisted around the suprapubic catheter. Cystogram confirmed the presence of a Foley balloon inside the bladder; there was no urinary fistula. The Foley balloon ruptured frequently, leading to extrusion of the Foley catheter. X-ray of abdomen showed heterotopic bone formation bridging the gap across diastasis of symphysis pubis. CT of pelvis revealed heterotopic bone lying in close proximity to the balloon of the Foley catheter; the sharp edge of heterotopic bone probably acted like a saw and led to frequent rupture of the balloon of the Foley catheter. Unique features of this case are: (1) temporal relationship of heterotopic bone formation to suprapubic cystostomy and chronic urine leak; (2) occurrence of heterotopic ossification in pubic region; (3) complications of heterotopic bone formation viz. frequent rupture of the balloon of the Foley catheter by the irregular margin of heterotopic bone and difficulty in insertion of suprapubic catheter because the heterotopic bone encroached on the suprapubic track; (4) synostosis between pubic bones as a result of heterotopic ossification..Common aetiological factors for neurogenic, heterotopic ossification, such as forceful manipulation, trauma, or spasticity, were absent in this patient. Since heterotopic bone formation was observed in the pubic region after suprapubic cystostomy and chronic urine leak, it is possible that risk factors related to the urinary tract might have played a role in heterotopic bone formation, which resulted in synostosis between pubic bones.

## INTRODUCTION

Neurogenic, heterotopic ossification is characterised by the formation of new, extraosseous (ectopic) bone in soft tissue surrounding peripheral joints in patients with neurological disorders[1]. Commonly reported predisposing factors for the occurrence of neurogenic, heterotopic bone formation include (1) genetic predisposition, (2) complete paralysis, (3) repeated forceful manipulation of paralysed limbs, (4) spasticity, (5) (micro) trauma, (6) deep venous thrombosis[1,2].

We report a female patient who was born with spina bifida, diastasis of symphysis pubis, and complete paraplegia, and developed neurogenic, heterotopic bone formation in the pubic region after undergoing suprapubic cystostomy.

## CASE REPORT

This female patient was born in 1973 with neural tube defect, diastasis of symphysis pubis, and complete paraplegia at T-10 level. She had been managing neuropathic bladder by indwelling urethral catheter. In 1996, she developed bypassing of the indwelling urethral catheter and was prescribed oxybutynin bladder instillations 5 mg, three times a day. This patient remained continent with oxybutynin bladder instillations until April 2000, when she developed bypassing of urethral catheter. X-ray of the pelvis was taken to look for stones in the urinary bladder. No radio-opaque vesical calculus was seen. Presence of diastasis of symphysis pubis with a wide gap between pubic bones was confirmed. (Fig. 1)

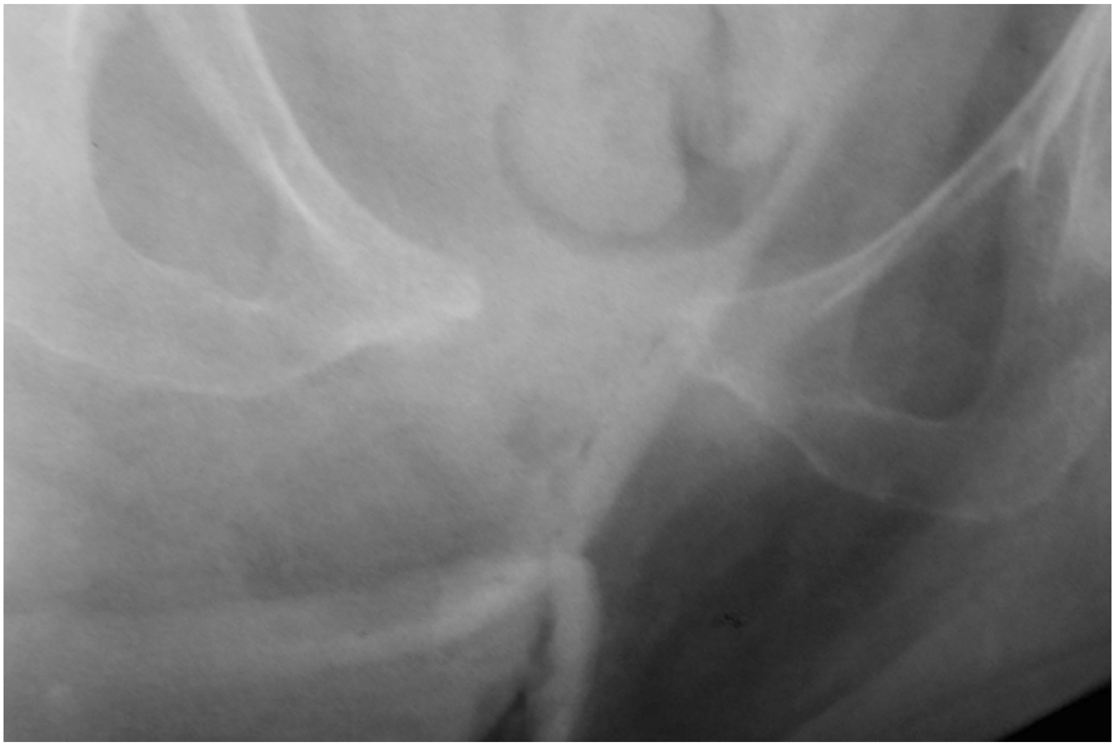

**FIGURE 1.** X-ray of pelvis, taken on 20 April 2000, shows no radio-opaque calculus in urinary bladder. Presence of diastasis of symphysis pubis with a wide gap between the pubic bones is confirmed.

In July 2000, she was prescribed diuretic, Burinex K (bumetanide 500 μg, potassium 7.7 mmol for modified release), for swelling of legs. Taking the diuretic tablet aggravated urine leak per urethra.

Therefore, she was prescribed tolterodine, 1 mg, twice a day. Since she continued to experience bypassing of urethral catheter, the dose of tolterodine was increased to 2 mg, three times a day in May 2001. In December 2001, suprapubic cystostomy was performed. A "Turner Warwick urethral staff" was inserted per urethra puncturing of the dome of the urinary bladder and then the anterior abdominal wall. A 20 Fr. Foley catheter was then guided into the urinary bladder. In February 2002, the suprapubic catheter was not working at all. There was bypassing around the suprapubic catheter, and she was leaking urine per urethra as well. In 2005, this patient developed sacral pressure sore, probably due to urine leak. X-ray of the abdomen was taken in May 2005 to look for urinary calculi; this X-ray showed no urinary calculi, but new bone formation was seen in the region of pubic diastasis. Heterotopic bone bridged the gap across diastasis of symphysis pubis. This patient had not developed fever; there was no soft tissue swelling or erythema over the pubic region.

Deroofing of pressure sore was done in July 2005. In September 2005, the urethra was mobilised and closed in four layers. Following closure of the urethra, there was no urine leak from the urethra, but urine leak persisted around the suprapubic catheter. Cystogram confirmed the presence of a Foley balloon inside the bladder; there was no urinary fistula. The Foley balloon ruptured frequently, leading to extrusion of the Foley catheter; this would occur as often as three times in 11 days. Further, health professionals experienced great difficulty in inserting a 14 Fr. all-silicone Foley catheter through the suprapubic track, but a stiff Nelaton catheter of size 10 or 12 Fr. could be pushed forcibly into the bladder. X-ray of the pelvis was taken to look for stones in the urinary bladder. There was no opaque calculus in the urinary bladder, but X-ray revealed that the heterotopic bone formation in the pubic region had resulted in synostosis between pubic bones whereas, previously, this patient had diastasis of symphysis pubis. The inferior margin of heterotopic bone was irregular especially on the left side (Fig. 2). On clinical examination, bone could be felt in the pubic region in midline, but there was no evidence of a lump, increased vascularity, or any other feature to suspect malignant change in the heterotopic bone.

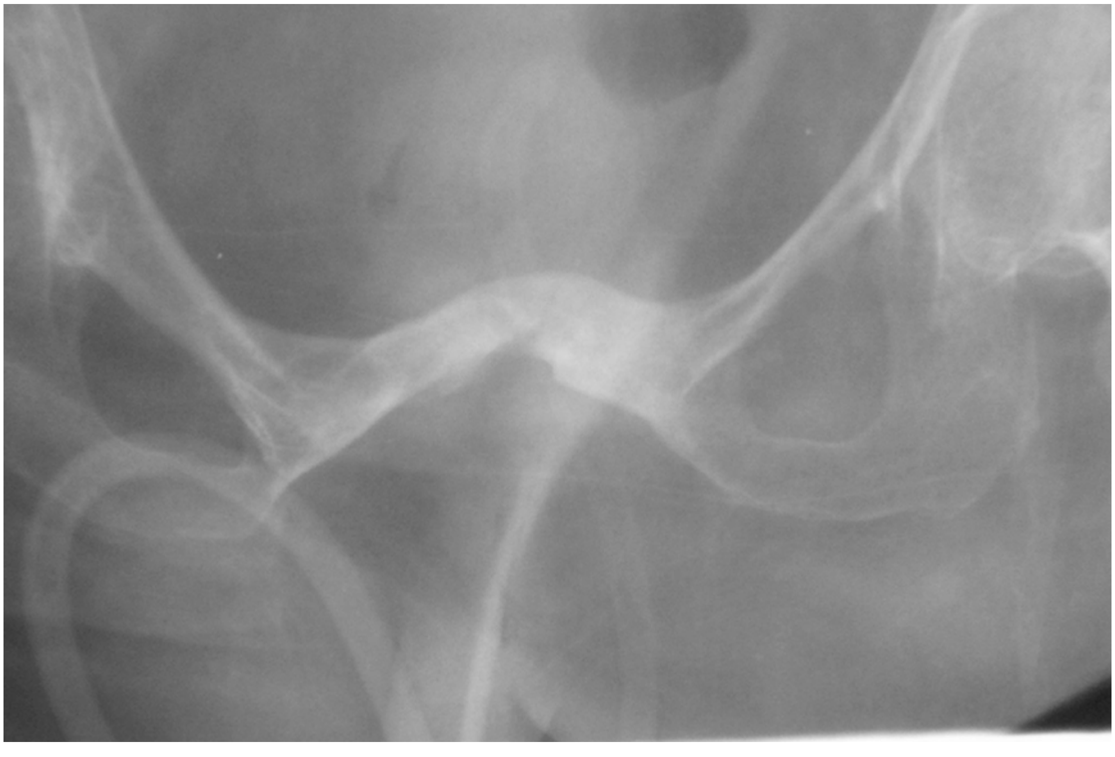

**FIGURE 2.** X-ray of pelvis, taken on 23 June 2006, shows heterotopic bone formation producing bony ankylosis of pubis in this patient, who was born with spina bifida and diastasis of symphysis pubis. Inferior margin of heterotopic bone, especially on the left side, is irregular.

Review of CT of the pelvis, performed in June 2006, revealed that an inferior edge of heterotopic bone had irregular margin and was lying adjacent to the balloon of the Foley catheter (Fig. 3). It was likely that the irregular edge of inferior margin of heterotopic bone lying in close proximity to the balloon of the Foley catheter acted almost like a saw, and led to frequent rupture of the balloon. The heterotopic bone was located very close to the track of suprapubic cystostomy, which would explain the difficulty in inserting a soft Foley catheter, whereas a stiff Nelaton catheter could be pushed forcibly into the bladder. This patient is currently awaiting ileal loop urinary diversion.

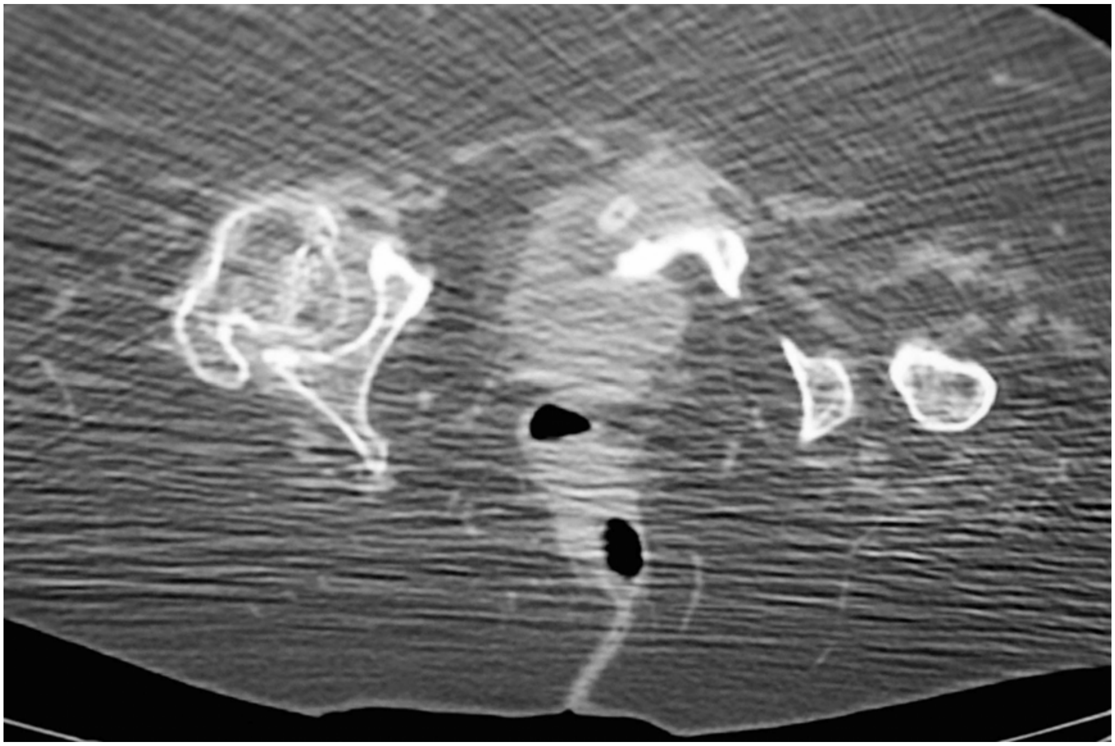

**FIGURE 3**. CT of pelvis (axial view) performed on 02 June 2006, shows cross-sectional view of Foley catheter and Foley balloon encircling the catheter. The sharp edge of heterotopic bone is seen abutting the balloon of Foley catheter from left side. Such close proximity of heterotopic bone with irregular spiky projections to the balloon of the Foley catheter might explain the frequent and spontaneous rupture of the balloon and subsequent extrusion of the suprapubic Foley catheter.

## DISCUSSION

Usually, heterotopic bone formation occurs in hip, knee, elbow, shoulder, hand, and spine (in decreasing incidence), but this patient developed heterotopic bone in the pubic region, which resulted in synostosis between the pubic bones. Further common clinical symptoms of heterotopic ossification, such as swelling, pain, erythema, warmth over the affected region, low-grade fever, and increased spasticity, were absent in this patient. Other unique features of this case are: (1) temporal relationship of heterotopic bone formation to suprapubic cystostomy and (2) urinary tract–related complications of heterotopic bone formation viz. frequent rupture of the balloon of the Foley catheter by the irregular margin of heterotopic bone and difficulty in insertion of suprapubic catheter as the heterotopic bone impinged on the suprapubic track.

Treatment of neurogenic, heterotopic ossification in persons with spinal cord injury includes disodium etidronate and nonsteroidal anti-inflammatory drugs (NSAIDs)[3]. Etidronate has mainly been used to block ectopic bone formation in the *early phase* after the clinical diagnosis of heterotopic ossification. For an optimal effect, etidronate should be started at an earlier phase of heterotopic ossification, before significant amounts of ectopic bone have been formed. In this patient, diagnosis of heterotopic ossification was made *after* mature bone had formed in the pubic region. Further, this patient did not develop any feature of inflammation and heterotopic ossification occurred silently after this patient underwent suprapubic cystostomy. Therefore, we could not have prescribed etidronate to slow down the mineralization process, or NSAIDs to inhibit the inflammatory process and suppress mesenchymal cell proliferation. Moreover, a substantive amendment to the Cochrane systematic review, made on 15 August 2004, states that given the absence of long-term radiographic outcomes in the included studies, there is insufficient evidence to recommend the use of disodium etidronate or other pharmacological agents for the treatment of acute heterotopic ossification[4].

In this patient, common predisposing factors for neurogenic, heterotopic ossification, such as forced passive movements, spasticity, or trauma, were absent. The site of occurrence of heterotopic bone was unusual in this patient; heterotopic bone formation was noted in the pubic region, which resulted in synostosis between pubic bones. Temporal sequence of events viz. suprapubic cystostomy, chronic urine leak, and then heterotopic bone formation in the pubic region, raises the possibility that risk factors related to the urinary system might be playing a role in heterotopic bone formation. Uncommon aetiological factors are: (1) an infected urinary tract serving as a source of antigenic material precipitating an immune response that triggers subsequent heterotopic ossification[5]; (2) inoculation of transitional epithelium into the soft tissues during suprapubic cystostomy, and subsequent heterotopic bone formation due to osteoinductive potential of human urothelium[6,7,8]; (3) chronic urine leak in the suprapubic region, which promoted heterotopic bone formation due to changes in local chemical and physical milieu[9].

## ACKNOWLEDGEMENT

Authors are grateful to Astra Tech Ltd, U.K., manufacturers of LoFric catheters, for generously providing financial support, enabling publication of this article under open access. Authors thank Dr. Subra for his valuable help in photography. Mr. P. Sett, Mr. G. Singh, Dr. C. Glass, Mrs. Marion Riley, Mrs. Jenny Bingley, and Mrs. Margaret Maule provided care to this patient.
